# Development and Clinical Validation of a Potential Penside Colorimetric Loop-Mediated Isothermal Amplification Assay of Porcine Circovirus Type 3

**DOI:** 10.3389/fmicb.2021.758064

**Published:** 2022-01-12

**Authors:** Jie Zhang, Miaomiao Li, Yunwen Ou, Danian Chen, Yaozhong Ding, Weibing Zhang, Yanjun Li, Qian Hou, Xiaoyun Li, Luoyi Zhou, Katarzyna Podgorska, Alexei D. Zaberezhny, Anna Szczotka-Bochniarz, Yongsheng Liu, Yang Wang

**Affiliations:** ^1^College of Animal Science and Technology, Hebei Normal University of Science and Technology, Qinhuangdao, China; ^2^State Key Laboratory of Veterinary Etiological Biology, Lanzhou Veterinary Research Institute, Chinese Academy of Agricultural Sciences, Lanzhou, China; ^3^Animal Disease Prevention and Control Center of Kaijiang County, Dazhou, China; ^4^Department of Swine Diseases, National Veterinary Research Institute, Puławy, Poland; ^5^Federal State Budgetary Institution, All-Russian Research and Technological Institute of Biological Industry (VNITIBP), Moscow, Russia

**Keywords:** vLAMP, PCV3, diagnosis, penside, one-step

## Abstract

Porcine circovirus type 3 (PCV3), a novel circovirus, imposes great burdens on the global pig industry. The penside tests for detecting PCV3 are critical for assessing the epidemiological status and working out disease prevention and control programs due to the unavailability of a commercial vaccine. A one-step molecular assay based on visual loop-mediated isothermal amplification (vLAMP) was developed for simple and rapid detection of PCV3. We compared its sensitivity and specificity with TaqMan quantitative real-time polymerase chain reaction (qPCR) and applied the developed assay in the epidemiological study of (*n* = 407) pooled swine sera collected from almost the entire mainland China during the years 2017–2018. We also explored the feasibility of the vLAMP assay for detecting raw samples without a prior DNA isolation step to expand its application capability. Results showed that the vLAMP assay could reliably detect the PCV3 cap gene with a detection limit of 10 DNA copies equal to that of the Taqman qPCR assay. In the epidemiological study, the PCV3 positive detection rate for 407 swine pooled sera detected by the vLAMP assay was 37.35% (152/407), whereas it was 39.01% (159/407) for Taqman qPCR. For the detection method without genome extraction, the results kept satisfactory specificity (100%) but displayed lower sensitivity (100% for CT < 32), indicating the direct detection is not sensitive enough to discriminate the samples with low viral loads. The one-step vLAMP is a convenient, rapid, and cost-effective diagnostic for penside detection and will enable the epidemiological surveillance of PCV3, which has widely spread in mainland China.

## Introduction

In 2016, a novel porcine circovirus [named porcine circovirus type 3 (PCV3)] distantly related to the known circoviruses was discovered to be associated with porcine dermatitis and nephropathy syndrome, reproductive failure (Palinski et al., 2017), and cardiac and multisystemic inflammation (Phan et al., 2016) in the United States. PCV3 was later proven to be widely prevalent in the swine population worldwide (Ku et al., 2017; Stadejek et al., 2017; Wang et al., 2017; Hayashi et al., 2018; Yuzhakov et al., 2018; Zoltán et al., 2019). Thus, this newly discovered DNA virus became an important threat to the healthy development of the pig industry. Furthermore, retrospective studies indicated that its first detection was traced back to 1996 (Fu et al., 2018; Loreto et al., 2018) or even millennia (Giovanni et al., 2019), according to the dataset analysis. In addition, PCV3 is very common in free-ranging pigs and wild boars (Dei Giudici et al., 2020) and was also found in the asymptomatic swine population (Zheng et al., 2017). As prevention strategies only targeting symptomatic animals are not enough to prevent the virus spread, the ability to rapidly, reliably, and cost-effectively diagnose PCV3 is crucial.

PCV3 is a member of *Porcine circoviruses* (PCVs), the smallest known DNA viruses in mammals, belonging to the *Circoviridae*. It is characterized by the non-enveloped and circular single-stranded DNA genome with around 2,000 nucleotides (nt). PCV3 contains three major open reading frames (ORFs): ORF1 encoding the Rep protein of 296 amino acid (aa), ORF2 editing the Cap protein of 214 aa, and ORF3 for a putative 231 amino acid (aa) protein of unknown roles. A recent study based on the largest datasets available of the full genome and ORF2 region formally suggested that PCV3 has only one genotype, “PCV3a” (Franzo et al., 2020), although different PCV3 classification schemes have been propounded (Li et al., 2018).

Up to now, there is no efficient vaccine available for PCV3. Therefore, rapid diagnostics of PCV3 with high sensitivity and specificity are critical for disease prevention and can help improve control programs, especially in the farms’ lack of professionals. Notably, penside diagnostics are vital to increasing the accessibility of testing around the world. There are several diagnostic assays for PCV3, including molecular and serological tests (Chen et al., 2018; Deng et al., 2018; Yang et al., 2019) though limited to the well-equipped laboratories due to their heavily relying on expensive lab equipment and professionally trained personnel. In contrast, loop-mediated isothermal amplification (LAMP) is a rapid and cost-effective molecular detection method (Notomi et al., 2000) that can amplify up to 10^9^ copies of target DNA sequences in a simple heat block or water bath within 1 h. The addition of indicator dyes can even enable the test results to be visually and qualitatively analyzed. Its rapidity, high sensitivity and specificity, and complex hardware independence make it an ideal method for penside diagnosis.

**FIGURE 1:**
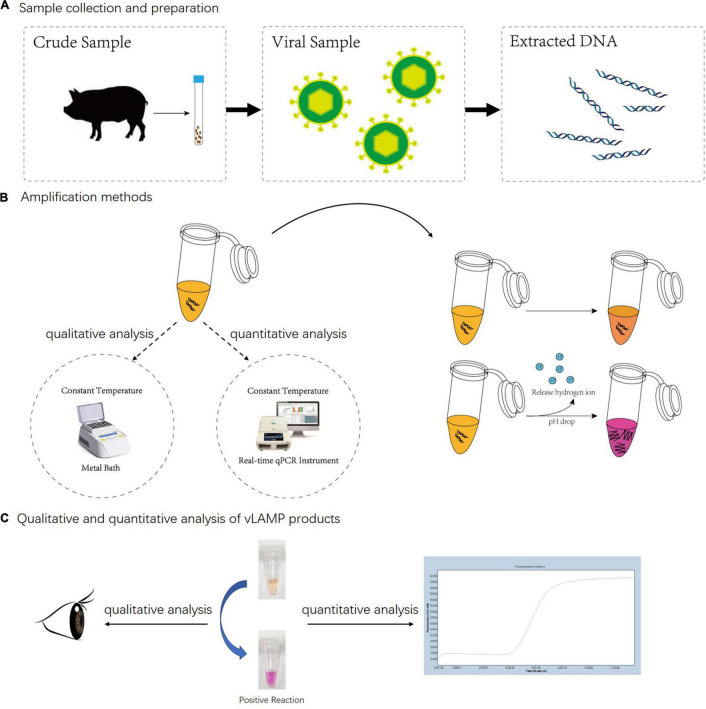
Whole detection procedure for vLAMP detection of PCV3. **(A)** Total DNA of PCV3 extracted from infected domestic pigs. **(B)** Metal bath and real-time qPCR instrument were used for qualitative and quantitative analyses separately. Neutral red chromogenic agents in reaction solutions led to color changes based on pH value as hydrogen ions released during DNA biosynthesis in positive reactions. For negative reaction, color of tube stayed same after reaction. **(C)** For qualitative analysis, results were directly discriminated with naked eye according to color changes, and for quantitative analysis, real-time qPCR was used to capture fluorescent signals; result was obtained from amplification curves.

In this study, to meet the visual detection, especially the penside test need for PCV3, we developed a one-step visual LAMP (vLAMP) assay to enable both qualitative and quantitative analyses by using the pH-dependent indicator dye (neutral red) (Figure 1). After successful amplifying of the highly conserved region of ORF2 by vLAMP, the key factors that influenced the amplification were optimized through response surface methodology (RSM). Then, the sensitivity of the vLAMP assay was assessed using the time to positive (T_*P*_) and cycle threshold (C_*T*_) as readouts for positive reactions of vLAMP and Taqman quantitative real-time polymerase chain reaction (qPCR) assay and correlated each other. The specificity was validated by detecting other common swine viruses usually causing clinical symptoms similar to PCV3. Furthermore, 407 pooled swine serum samples collected during 2017–2018 from six selected geographical regions representing almost the entire mainland China were subjected to the detection of PCV3 vLAMP and Taqman qPCR. To some extent, detecting the pooled specimens could better reflect the true situation, for it is too expensive for farmers to test every animal individually. Moreover, we also developed a direct-detection method by using the crude serum sample as the amplification template without a regular DNA extraction procedure. We tested 48 pooled serum samples covering a broad range of viral loads, enabling us to precisely confirm its sensitivity.

## Materials and Methods

### Phylogenetic Analysis

A total of 2,431 DNA sequences were retrieved from the National Center for Biotechnology Information using BLASTn with KY778777.1 reference sequence as the query, from which 1,379 sequences were specific for PCV3, and the rest were relevant to other species. Full-length PCV3 sequences were collated (*n* = 654) and aligned using the ClustalW in MEGA7.0 (Kumar et al., 2016) with KY778777.1 as the reference. The phylogenetic tree was then built using a neighbor-joining approach based on the alignment of 97 unique sequences specific for PCV3 with KY778777.1 as a reference. The detailed information is listed in Supplementary Figure 1.

### Primer Design

The *cap* gene is highly conserved and is often used for all kinds of PCR detection of PCV3 (Palinski et al., 2017; Wang et al., 2017). A set of six candidate vLAMP primers targeting eight different fragments were designed using the Primer Explorer 5.0^1^ and MEGA7.0 (Kumar et al., 2016) based on the multiple sequence alignments and phylogenetic analysis of 97 PCV3 *cap* sequences retrieved from the National Center for Biotechnology Information. All primers were commercially purchased (Tsingke Biotechnology Co., Ltd., Xian, China).

### Optimization of Incubation Temperature and Time

The incubation temperature and time were the key factors affecting the amplification. Therefore, they were optimized through RSM. Different parameters of reaction temperature and incubation time were tested to explore the response of fluorescence units (FUs). FU was calculated using a Real-Time Thermal Cycler (Roche, LightCycler^®^ 480II). The parameters of factors and response values are shown in Supplementary Table 1.

### Viruses and Viral DNA/RNA

PCV3, PCV2, genotype II African swine fever virus, Japanese encephalitis virus, classical swine fever virus, porcine reproductive and respiratory syndrome, pseudorabies virus, porcine parvovirus, porcine epidemic diarrhea virus, swine influenza virus, and non-pathogenic PCV1 were all kept at −70°C in Lanzhou Veterinary Research Institute, Chinese Academy of Agricultural Sciences. All viral genomes were obtained from cell cultures following the instructions of MiniBEST Viral RNA/DNA Extraction Kit ver.5.0 (Takara, Code No. 9766).

### Clinical Sample Handling for Visual Loop-Mediated Isothermal Amplification Detection

One thousand four hundred two clinical serum specimens were collected in the whole mainland China during 2017–2018 using sterile tools and transferred to 1.5-ml centrifuge tubes. Three to five specimens from the same area were mixed into one, and 407 pooled swine sera were obtained. Genomes were isolated using MiniBEST Viral RNA/DNA Extraction Kit ver.5.0 (Takara, Code No. 9766), according to the instructions of the kit. All samples were subjected to both Taqman qPCR and vLAMP assay.

### Preparation of a Plasmid Standard

The pUC57-PCV3 containing the whole genome of PCV3 was synthesized by the Genscript Biotech Corporation (Nanjing, China) according to the sequences released in GenBank (accession number KY778777.1). A sensitivity assay of vLAMP was created by 10-fold diluting the purified pUC57-PCV3 plasmid in double-distilled water (ddH_2_O) from 10^7^ to 10^0^ copies/μl.

### Taqman Quantitative Real-Time Polymerase Chain Reaction Assay

TaqMan qPCR, as described previously (Wang et al., 2017), which was compared with both conventional PCR and qPCR (Ku et al., 2017; Palinski et al., 2017), was performed with minor modifications to compare its sensitivity and specificity with that of the vLAMP assay developed in our study. Briefly, the qPCR was performed in a final volume of 20 μl comprising ddH_2_O (7.5 μl), 2× qPCR mixture (10 μl), 50-pmol sense primer (1.0 μl), 50-pmol antisense primers (1.0 μl), and 5-pmol probes (1.0 μl) and 2 μl of the template in a Real-Time Thermal Cycler (Bio-rad CFX96) under the following conditions: 95°C for 15 min and 45 cycles of 94°C for 15 s and 60°C for 1 min. C_*T*_ values below the cutoff value (C_*T*_ < 40) were considered positive.

### Visual Loop-Mediated Isothermal Amplification Assay

The reaction system of vLAMP included 1-μl 8.0 U BST DNA polymerase (New England Biolabs Inc.) and 1-μl template DNA with 18 μl of reaction buffer [50-mM KCl, 10-mM (NH_4_)_2_SO_4_, 25-μM neutral red, 8-mM MgSO_4_, 0.1% Tween 20, 0.5-M betaine, 1.4-mM dNTP, 0.08-μM F3 primer, 0.08-μM B3 primer, 0.8-μM FIP primer, 0.8-μM BIP primer, 0.2-μM LF primer, and 0.2-μM LB primer]. The LAMP reaction was carried out at 60°C for 60 min.

Qualitative and quantitative analyses of vLAMP products:

For quantitative analysis, a Real-Time Thermal Cycler (Bio-rad CFX96) was used to capture the fluorescent signals. The time to positive (T_*P*_) was determined when reactions achieved a fluorescence threshold above the control.

Metal bath is enough for qualitative detection. The experimental results for color recognition were directly observed with the naked eyes. After the reaction finished, tubes with a pink color indicated successful LAMP amplification, whereas orange indicated negative samples. The amplicons could also be detected by electrophoresis on the 2% agarose gel to observe the formation of the specific ladder- like bands.

### Digestion of Visual Loop-Mediated Isothermal Amplification Products With Restriction Enzymes and Sequencing

One-microgram vLAMP products were digested by adding 2-μl 10× FastDigest buffer and 1-μl Eco47III (Thermo Fisher, Code No. #FD0324) as well as sterile and distilled water to bring the reaction mixtures to 20 μl. The reaction was performed in a PCR amplifier at 37°C for 5 min, and the amplification products were then analyzed on a 2% agarose gel by electrophoresis and subsequently sequenced by Tsingke Biotechnology Co., Ltd., Xian, China.

### Data Analysis

Graphs were all graphed using GraphPad Software Inc., Prism Version 8, United States. For all comparative analyses, the Taqman qPCR was used as the gold standard. All reactions were repeated at least once and were generally carried out in triplicate.

## Results

### Phylogenetic Analysis and Validation of the Visual Loop-Mediated Isothermal Amplification Primers

**FIGURE 2:**
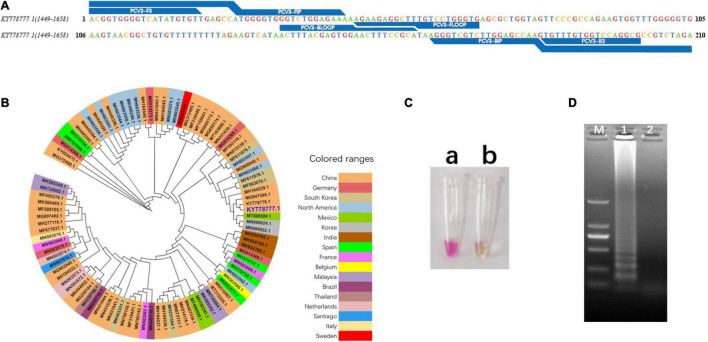
Design and validation of vLAMP primers. **(A)** Target region of vLAMP primers is shown, and reference sequence is KY778777.1 PCV3. **(B)** Phylogenetic tree of PCV3 complete genome. Phylogenetic analysis was carried out to explain universality of reference sequence. Strains from different countries are marked with different colors, and reference sequence KY778777.1 is marked in large dark blue font. **(C)** Validation of vLAMP primers. Color change proved good performance of vLAMP primers. (a) A pink color occurred in vLAMP reaction with 10^6^ copies/μl pUC57-PCV3, as templates indicated a positive reaction; (b) A orange color occurred in vLAMP with ddH_2_O, as templates indicated a negative reaction. **(D)** Gel electrophoresis of vLAMP products. Ladder-like bands indicated success amplification. From left to right: lane M, DNA Marker DL-2000 (Takara); lane 1, vLAMP with 10^6^ copies/μl pUC57-PCV3 as templates; lane 2, vLAMP with ddH_2_O as templates.

The primer sets used in this study were designed based on phylogenetic analysis and alignment of PCV3 genomic sequences (Figure 2B and Supplementary Figure 1). Primer sequences and locations can be found in Table 1 and Figure 2A. The phylogenetic analysis showed that the nucleotide homology of PCV3 strain was high (>97%), which indicated the vLAMP primers designed here could theoretically target the vast majority of PCV3 in the world. To estimate the validity of the vLAMP primers, vLAMP assay was then performed, and the products were visualized by both naked eyes and 1.2% agarose gel electrophoresis. After the reaction finished, an expected color shift occurred and the typical ladder-like pattern of DNA concatemers (Figures 2C,D), which indicated the vLAMP reaction ran normally. To further confirm the specificity of the amplification, the vLAMP products were then digested by Eco47III and sequenced (Supplementary Figure 2). All results showed that the PCV3 vLAMP primers could effectively amplify the target gene.

**TABLE 1:** Nucleotide sequences of primers used in vLAMP assay.

Primer	Type	Position^a^	Sequence (5′–3′)^b^
PCV3-F3	Forward outer	1,449–1,468	ACGGTGG**GGTC**ATATGTGTT
PCV3-B3	Reverse outer	1,649–1,632	CGCCTGGACCACAAACAC
PCV3-FIP (F1c+TTTT+F2)	Forward inner	1,543–1,522, 1,475–1,493	CCAATTCTGGCGGGAACTACCA**TTTT** TGGGGTGGGTCTGGAGAAA
PCV3-BIP (B1c+TTTT+B2)	Reverse inner	1,548–1,570, 1,631–1,613	GGGGTGAAGTAACGGCTGTGTTT**TTTT** TTGGCTCCAAGACGACCCT
PCV3-FLOOP	Forward Loop	1,516–1,495	CACCCAGGACAAAGCCTCTTCT
PCV3-BLOOP	Reverse Loop	1,588–1,611	CTTTACGAGTGGAACTTTCCGCAT

*^a^Position numbers are based on complete genome of PCV3/CN/Shandong-2/201703 strain (GenBank accession number KY778777.1).*

*^b^Boldface indicates TTTT spacer between F1c and F2 or B1c and B2.*

### Optimization of the Visual Loop-Mediated Isothermal Amplification Reaction

**FIGURE 3:**
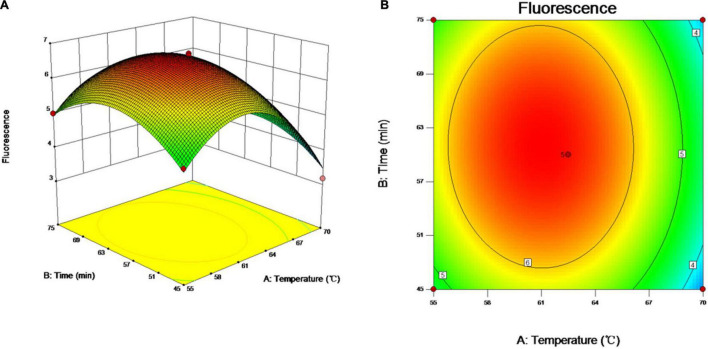
Optimization of vLAMP reaction. Three- and two-dimensional plots of effect of interactions between fluorescence units (FUs) and variables (incubation time and reaction temperature). These plots are depiction of response surface methodology by central composite design. Minimum–maximum points of incubation time and reaction temperature that we designed were 45–75 min and 55–70°C, 13 different combinations were supplied by Design-Expert software, and FU values of different combinations obtained from real-time qPCR experiments. **(A)** Three-dimensional plots of effect of interactions between FU and incubation time and reaction temperature. **(B)** Two-dimensional plots of effect of interactions between FU and incubation time and reaction temperature.

As incubation temperature and reaction time are the key factors affecting the quality of vLAMP products as well as RSM helping identify the combination of two-factor levels to achieve the optimal response (Chen et al., 2021), therefore, the two essential factors influencing the efficiency of amplification were optimized by RSM in the study. The relationship between the responses of FUs and the experimental variables was illustrated graphically by plotting both the response values vs. the experimental factor values simultaneously (Figure 3). The results showed that the FU increased with reaction time and incubation temperature until an equilibrium was reached. The FU decreased later beyond the optimal value of these variables. The data showed that the maximum FU could be achieved with 60 min of reaction time and 60°C of incubation temperature.

### Analytical Sensitivity and Specificity of the Visual Loop-Mediated Isothermal Amplification Assay

**FIGURE 4:**
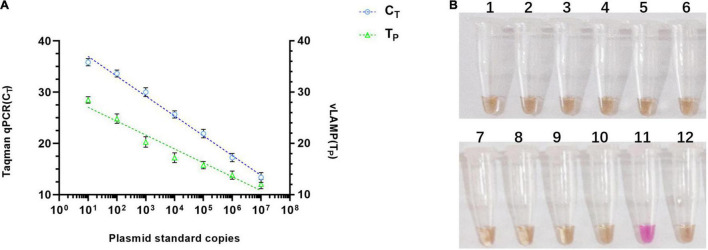
Sensitivity and specificity of vLAMP assay. **(A)** Sensitivity comparison between established vLAMP and Taqman qPCR assay from plasmid standards as templates showed equal sensitivity of these two methods indicated that they revealed a similar lower limit of detection. **(B)** Specificity test showed good specificity of established vLAMP assay. Genomes of different viruses were added to vLAMP reaction and incubated at 60°C for 60 min. Order is as follows: (1) PCV1, (2) PCV2, (3) genotype II African swine fever virus, (4) Japanese encephalitis virus, (5) classical swine fever virus, (6) porcine reproductive and respiratory syndrome, (7) pseudorabies virus, (8) porcine parvovirus, (9) porcine epidemic diarrhea virus, (10) swine influenza virus, (11) 10^6^ copies/μl pUC57-PCV3, and (12) ddH_2_O. Only sample 11 (PCV3) was positive.

To validate the property of the proposed vLAMP assay, the analytical sensitivity and specificity were tested. As shown in Figure 4A, the range of T_*P*_ values was 11–29 min, with 10 copies as the limit of detection in the vLAMP reaction roughly equivalent to that of the Taqman qPCR. Furthermore, all viruses kept in the laboratory used to assess the analytical specificity of the vLAMP were confirmed to guarantee the reliability of those viral nucleic acids by self-identification with the standard PCR or qPCR (Supplementary Figure 3). No cross-reactions were observed with PCV1, PCV2, genotype II African swine fever virus, Japanese encephalitis virus, classical swine fever virus, porcine reproductive and respiratory syndrome, pseudorabies virus, porcine parvovirus, porcine epidemic diarrhea virus, and swine influenza virus (Figure 4B), indicating the high specificity of our PCV3 vLAMP.

### Performance Comparison of the Porcine Circovirus Type 3 Visual Loop-Mediated Isothermal Amplification and Taqman qPCR With Clinical Samples

**FIGURE 5:**
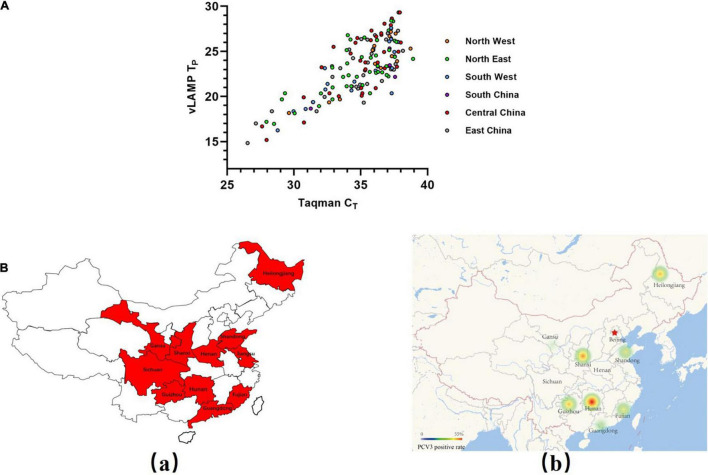
Evaluation of vLAMP assay with clinical serum samples. **(A)** Detection results of a total of 407 pooled swine serum samples from six selected geographical regions of China using both Taqman qPCR and vLAMP were shown by scatter plot. X-axis showed C_*T*_ values of TaqMan qPCR assay, and Y-axis showed T_*P*_ values of vLAMP assay. **(B)** Swine serum sample collection areas in this study and distribution of PCV3 detected by vLAMP in China. (a) Sample collection areas in this study. (b) Thermal map based on a positive rate of PCV3.

Four hundred seven pooled swine serum samples collected from six selected geographical regions (total 7) representing almost the entire mainland China during 2017–2018 were tested with the newly established vLAMP and Taqman qPCR assay, respectively. The viral DNA concentrations based on the Taqman qPCR standard curve are detailed in Supplementary Table 2. All these clinical samples could be divided into four categories (strongly positive, positive, weak positive, and negative) based on cycle threshold (C_*T*_) values (Table 2). Results obtained with the vLAMP and Taqman qPCR are shown in Figure 5A, Table 3, and Supplementary Table 2. The vLAMP assay showed sensitivity and specificity of 95.60 and 100%, respectively. From the 407 samples, the vLAMP diagnosed 255 samples as negative and 152 as positive, showing 98.28% concordance with Taqman qPCR. The results also indicated that PCV3 was already widespread in China during 2017–2018 (Figure 5B).

**TABLE 2:** Categories and characteristics of clinical samples tested in this study.

cat.^a^	Taqman qPCR C_T_ range (cycles)	DNA conc.^b^ (copies/μl)	samples (n)
SP	<30	>10^3^	12
P	30–34.99	10^3^ to 50	47
WP	34.99–39.99	50 to 1	100
N	>40; NEG	/	248
Total	/	/	407

*^a^Clinical samples are classified into categories (cat.) based on Taqman qPCR C_T_ values; SP, strong positive; P, positive; WP, weakly positive; N, negative.*

*^b^DNA estimated concentration (copies/μl) based on Taqman qPCR standard curve.*

**TABLE 3:** Sensitivity and specificity of vLAMP VS Taqman qPCR for PCV3 detection.

Assay	Taqman qPCR positive (n)	Taqman qPCR negative (n)	Sensitivity^a^ (%)	Specificity^b^ (%)
vLAMP positive (n)	152	0	95.60	100
vLAMP negative (n)	7	248	95.60	100

*^a^Sensitivity calculated based on SEN (%) = TP / (TP + FN) × 100.*

*^b^Specificity calculated based on SPE (%) = TN / (TN + FP) × 100.*

### Porcine Circovirus Type 3 Direct-Detection Visual Loop-Mediated Isothermal Amplification Free of DNA Isolation

**FIGURE 6:**
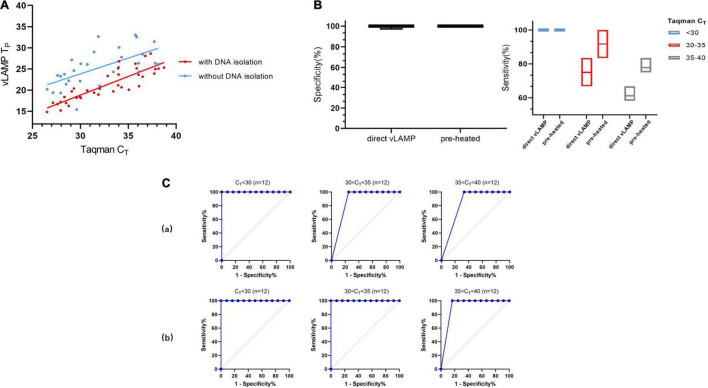
Evaluation of direct vLAMP assay with clinical serum samples. **(A)** Forty-eight clinical serum samples (36 positives and 12 negatives) covering all sample types were subject to either vLAMP (red plot) or direct vLAMP assay (blue plot). Result shows most samples with a C_*T*_ < 32 could be detected by both vLAMP and direct-vLAMP assay. **(B)** Evaluation of sensitivity and specificity of direct-vLAMP and preheated direct-vLAMP assay. To investigate if preheated serum samples could influence results, 48 serum samples were selected to evaluate specificity and sensitivity of vLAMP assay after preheated handling. Left shows that preheated have little influence on specificity of vLAMP, whereas right shows a higher sensitivity after preheated especially when samples’ C_*T*_ ≥ 30. **(C)** Receiver operating characteristic (ROC) curves analysis to indicate accuracy of direct-vLAMP and preheated direct-vLAMP assay on samples of different C_*T*_ ranges. When C_*T*_ value of samples was less than 35, preheated increased sensitivity strikingly. (a) ROC curves of direct-vLAMP assay. (b) ROC curves of preheated direct-vLAMP assay.

Based on the experiments described earlier, we decided to detect the serum samples without any treatment to expand their practicability. We chose 48 serum samples that represented all sample types described in Table 2 for the direct-detection vLAMP. The direct-detection assay could yield a positive reaction in the majority of samples with a C_*T*_ < 32 (Figure 6A and Supplementary Table 3). Some studies showed that (Dao Thi et al., 2020; Wang et al., 2021) heat treatment of clinical samples enables vLAMP assay a higher sensitivity. Thus, we preheated the serum samples at 95°C for 10 min and collected the supernate for detection. The results showed that the improved method maintained the specificity and exhibited a higher sensitivity with a C_*T*_ < 35 (Figure 6B). We further validated these samples with Taqman qPCR and sequenced them, and the results proved the presence of the PCV3 gene (Supplementary Figure 4). The direct-detection vLAMP could not sense weak positive samples (C_*T*_ > 35), indicating its deficiency in discriminating low viral load samples. However, the receiver operating characteristic curve analysis showed the method had better accuracy in the detection of samples with a C_*T*_ value less than 35 (Figure 6C).

## Discussion

In this study, a vLAMP assay, enabling both qualitative and quantitative analyses for PCV3, was established. We could both identify the results of the reactions with naked eyes and use a T_*P*_ value to analyze the results quantitatively. The T_*P*_ value could also be used as a reference for analyzing the virus loads of the sample, just like the C_*T*_ value in Taqman qPCR. The assay was specific and had a limit of detection of 10 copies per reaction, which was the same as Taqman qPCR. This proposed assay is featured by the simplicity of operator and lab equipment independence. As the positive results could be identified by an obvious color change, we therefore would recommend the qualitative analysis. Furthermore, determining results without opening reaction tubes could effectively reduce the possibility of contamination. Although there are several diagnosis assays, including molecular detection methods such as conventional and real-time qPCR and serological tests (for example, enzyme-linked immunosorbent assay), these diagnostics are required to run on the specific instruments and/or to be operated by skilled persons in the laboratory settings. Therefore, such assays are very hard to be applied in the less-developed local labs and even impossible for the penside test. In this sense, the vLAMP test developed in this study may supply a portable and affordable solution (approach) for the rapid identification of PCV3. The clinical application of this established method showed a widespread of PCV3 in China continent, indicating wide application prospects of this method.

To further improve the convenience of analysis, we explored the feasibility of detecting the crude samples without DNA isolation. Despite being faster and more convenient, the direct detection assay impaired the sensitivity, indicating the method was not adapted to discriminate low viral load samples. In addition, we also found that heat treatment could stably improve the sensitivity of the direct detection vLAMP assay. The preheat likely helped inactivate DNAses and to break up the viral capsid to release the viral DNA without destroying the DNA. In conclusion, our study presented the possibility of using a direct detection vLAMP test in undeveloped areas.

Although the proposed vLAMP assay is more portable than qPCR and the serological tests described before (Chen et al., 2018; Deng et al., 2018; Yang et al., 2019), there is still a huge space for improvement for our work, for example, the temperature limitation of preservation and transportation of the vLAMP reagents. To overcome this limitation, a reagent in the form of dry powder should be developed. In addition, the micro-quantification of the reaction system can greatly reduce the cost. To further increase the portability, a chip device that could sense and process the signal changes caused by amplification during the reaction is needed. The device could enable detection of results, visualization, and geolocalization when paired with a smartphone application.

In conclusion, our study showed that PCV3 has widespread in China. The vLAMP assay developed here is a powerful tool for case identification and epidemiological surveillance of PCV3, especially in underdeveloped areas.

## Data Availability Statement

The original contributions presented in the study are included in the article/Supplementary Material, further inquiries can be directed to the corresponding author/s.

## Author Contributions

YW, YoL, AS-B, and JZ supported and supervised the whole study. JZ, YW, and ML designed the study, analyzed the data, and drafted the manuscript. YO, DC, YD, WZ, YaL, QH, XL, and LZ were responsible for acquiring the data. KP and AZ revised the manuscript. All authors contributed to the article and approved the submitted version.

## Conflict of Interest

The authors declare that the research was conducted in the absence of any commercial or financial relationships that could be construed as a potential conflict of interest.

## Publisher’s Note

All claims expressed in this article are solely those of the authors and do not necessarily represent those of their affiliated organizations, or those of the publisher, the editors and the reviewers. Any product that may be evaluated in this article, or claim that may be made by its manufacturer, is not guaranteed or endorsed by the publisher.

## Footnotes

1
http://primerexplorer.jp/lampv5e/index.html

## Supplementary Material

Supplementary Figure 1 Alignment of unique sequences retrieved from the blast query in NCBI (*n* = 97) with KY778777.1 as a reference for PCV3. The cap gene sequences covered by vLAMP primers was displayed.

Supplementary Figure 2 Results of restriction endonuclease digestion and sequencing of vLAMP products. **(A)** Agarose gel electrophoresis shows vLAMP products after Eco47III digestion (lane 2), the expected band size after restriction endonuclease digestion is 140, 167, and 261 bp. Undigested vLAMP reaction product is also shown for comparison (lane 1). Lane M, DNA Marker DL-2000 (Takara). **(B)** Sequence Alignment shows the sequencing results of the 139 bp enzyme digestion products in A. The alignment indicated the identity was 97.12% (135/139), except for 4 nucleotides mismatching the reference sequences due to the sequencing error.

Supplementary Figure 3 Self-identification of CSFV, PRRSV, PRV, PEDV, JPEV, PCV1, PCV2, ASFV, PPV, and SIV. **(A)** Real-time qPCR experiments shows the self-identification results. The ddH_2_O was amplified as the NC in these detection. (a) JEPV, (b) PCV1, (c) PCV2, (d) ASFV, (e) PPV, (f) SIV. **(B)** Conventional PCR methods and agarose gel electrophoresis show the self-identification results. The expected band size of each virus are 400 bp (PRRSV), 217 bp (PRV), 671 bp (CSFV), 272 bp (CSFV) and 315 bp (PEDV), respectively. (a) PRRSV, (b) PRV, (c) and (d) CSFV, (e) PEDV. For each result, Lane 1, DNA marker; Lane 2, the PCR products; Lane 3, NC.

Supplementary Figure 4 Results of validation of the presence of PCV3 in samples (Ct > 32) by Taqman qPCR. The sequencing result of the 78 bp Taqman qPCR products using samples whose Ct > 32. The alignment indicated the identity was 98.7% (78/78), except for 1 nucleotides mismatching the reference sequences due to sequencing primer.
