# Supplementary material for: Development and Clinical Validation of a Potential Penside Colorimetric Loop-Mediated Isothermal Amplification Assay of Porcine Circovirus Type 3
Source: Front Microbiol. 2022 Jan 12;12:758064. doi: 10.3389/fmicb.2021.758064 (PMC8790240; doi:10.3389/fmicb.2021.758064)
Supplement: Supplementary file 1 [file Table_1.DOCX]

| A: Temperature (℃) | B: Time (min) | Response Fluorescence Unit |
| --- | --- | --- |
| 62.50 | 81.21 | 5.13 |
| 62.50 | 38.78 | 4.78 |
| 55.00 | 75.00 | 5.01 |
| 62.50 | 60.00 | 6.68 |
| 70.00 | 75.00 | 3.27 |
| 55.00 | 45.00 | 4.78 |
| 62.50 | 60.00 | 6.74 |
| 73.10 | 60.00 | 3.01 |
| 62.50 | 60.00 | 6.62 |
| 62.50 | 60.00 | 6.67 |
| 70.00 | 45.00 | 3.18 |
| 62.50 | 60.00 | 6.69 |
| 51.89 | 60.00 | 4.28 |

Table S1. Response surface analysis scheme and response fluorescence unit values.
