# Supplementary material for: Development and Clinical Validation of a Potential Penside Colorimetric Loop-Mediated Isothermal Amplification Assay of Porcine Circovirus Type 3
Source: Front Microbiol. 2022 Jan 12;12:758064. doi: 10.3389/fmicb.2021.758064 (PMC8790240; doi:10.3389/fmicb.2021.758064)
Supplement: Supplementary file 2 [file Table_2.DOCX]

**Table S2. Detection results of pooled swine serum samples using Taqman qPCR and vLAMP**

| No. | Sample origin | Taqman qPCR C_T_ | vLAMP  T_P_ | DNA conc.^a^ (copies/µl) | cat.^b^ |
| --- | --- | --- | --- | --- | --- |
|  | North West | NO C_T_ | NO T_P_ | / | N |
|  | North West | 29.62 | 18.17 | >10^3^ | SP |
|  | North West | 33.44 | 19.69 | 1 to 10^3^ | P |
|  | North West | NO C_T_ | NO T_P_ | / | N |
|  | North West | 36.02 | 25.61 | 1 to 50 | WP |
|  | North West | NO C_T_ | NO T_P_ | / | N |
|  | North West | NO C_T_ | NO T_P_ | / | N |
|  | North West | 36.50 | 23.17 | 1 to 50 | WP |
|  | North West | 38.73 | 25.31 | 1 to 50 | WP |
|  | North West | NO C_T_ | NO T_P_ | / | N |
|  | North West | 32.63 | 19.36 | 1 to 10^3^ | P |
|  | North West | NO C_T_ | NO T_P_ | / | N |
|  | North West | 35.88 | 25.14 | 1 to 50 | WP |
|  | North West | 37.15 | 26.69 | 1 to 50 | WP |
|  | North West | NO C_T_ | NO T_P_ | / | N |
|  | North West | 37.25 | 27.17 | 1 to 50 | WP |
|  | North West | 37.61 | 26.98 | 1 to 50 | WP |
|  | North West | 36.07 | 23.63 | 1 to 50 | WP |
|  | North West | 37.33 | 24.47 | 1 to 50 | WP |
|  | North West | NO C_T_ | NO T_P_ | / | N |
|  | North West | 37.72 | 23.91 | 1 to 50 | WP |
|  | North West | NO C_T_ | NO T_P_ | / | N |
|  | North West | NO C_T_ | NO T_P_ | / | N |
|  | North West | NO C_T_ | NO T_P_ | / | N |
|  | North West | NO C_T_ | NO T_P_ | / | N |
|  | North West | NO C_T_ | NO T_P_ | / | N |
|  | North West | NO C_T_ | NO T_P_ | / | N |
|  | North West | NO C_T_ | NO T_P_ | / | N |
|  | North West | NO C_T_ | NO T_P_ | / | N |
|  | North West | NO C_T_ | NO T_P_ | / | N |
|  | North West | NO C_T_ | NO T_P_ | / | N |
|  | North West | NO C_T_ | NO T_P_ | / | N |
|  | North West | NO C_T_ | NO T_P_ | / | N |
|  | North West | NO C_T_ | NO T_P_ | / | N |
|  | North West | NO C_T_ | NO T_P_ | / | N |
|  | North West | NO C_T_ | NO T_P_ | / | N |
|  | North West | NO C_T_ | NO T_P_ | / | N |
|  | North West | NO C_T_ | NO T_P_ | / | N |
|  | North East | NO C_T_ | NO T_P_ | / | N |
|  | North East | NO C_T_ | NO T_P_ | / | N |
|  | North East | NO C_T_ | NO T_P_ | / | N |
|  | North East | NO C_T_ | NO T_P_ | / | N |
|  | North East | NO C_T_ | NO T_P_ | / | N |
|  | North East | NO C_T_ | NO T_P_ | / | N |
|  | North East | NO C_T_ | NO T_P_ | / | N |
|  | North East | 36.15 | 22.62 | 1 to 50 | WP |
|  | North East | 37.55 | 26.18 | 1 to 50 | WP |
|  | North East | 36.14 | 24.54 | 1 to 50 | WP |
|  | North East | NO C_T_ | NO T_P_ | / | N |
|  | North East | 37.43 | 28.34 | 1 to 50 | WP |
|  | North East | NO C_T_ | NO T_P_ | / | N |
|  | North East | NO C_T_ | NO T_P_ | / | N |
|  | North East | NO C_T_ | NO T_P_ | / | N |
|  | North East | NO C_T_ | NO T_P_ | / | N |
|  | North East | 35.13 | 21.26 | 1 to 50 | WP |
|  | North East | NO C_T_ | NO T_P_ | / | N |
|  | North East | NO C_T_ | NO T_P_ | / | N |
|  | North East | NO C_T_ | NO T_P_ | / | N |
|  | North East | NO C_T_ | NO T_P_ | / | N |
|  | North East | NO C_T_ | NO T_P_ | / | N |
|  | North East | 34.03 | 26.80 | 1 to 10^3^ | P |
|  | North East | NO C_T_ | NO T_P_ | / | N |
|  | North East | NO C_T_ | NO T_P_ | / | N |
|  | North East | 33.99 | 23.67 | 1 to 10^3^ | P |
|  | North East | NO C_T_ | NO T_P_ | / | N |
|  | North East | NO C_T_ | NO T_P_ | / | N |
|  | North East | NO C_T_ | NO T_P_ | / | N |
|  | North East | NO C_T_ | NO T_P_ | / | N |
|  | North East | NO C_T_ | NO T_P_ | / | N |
|  | North East | NO C_T_ | NO T_P_ | / | N |
|  | North East | NO C_T_ | NO T_P_ | / | N |
|  | North East | NO C_T_ | NO T_P_ | / | N |
|  | North East | NO C_T_ | NO T_P_ | / | N |
|  | North East | NO C_T_ | NO T_P_ | / | N |
|  | North East | NO C_T_ | NO T_P_ | / | N |
|  | North East | NO C_T_ | NO T_P_ | / | N |
|  | North East | NO C_T_ | NO T_P_ | / | N |
|  | North East | NO C_T_ | NO T_P_ | / | N |
|  | North East | 37.13 | 25.14 | 1 to 50 | WP |
|  | North East | NO C_T_ | NO T_P_ | / | N |
|  | North East | NO C_T_ | NO T_P_ | / | N |
|  | North East | NO C_T_ | NO T_P_ | / | N |
|  | North East | NO C_T_ | NO T_P_ | / | N |
|  | North East | 37.07 | 22.24 | 1 to 50 | WP |
|  | North East | NO C_T_ | NO T_P_ | / | N |
|  | North East | 37.89 | NO T_P_ | 1 to 50 | WP |
|  | North East | 36.22 | 26.25 | 1 to 50 | WP |
|  | North East | 35.75 | 24.30 | 1 to 50 | WP |
|  | North East | NO C_T_ | NO T_P_ | / | N |
|  | North East | NO C_T_ | NO T_P_ | / | N |
|  | North East | 34.71 | 22.19 | 1 to 10^3^ | P |
|  | North East | NO C_T_ | NO T_P_ | / | N |
|  | North East | NO C_T_ | NO T_P_ | / | N |
|  | North East | 37.67 | 26.06 | 1 to 50 | WP |
|  | North East | NO C_T_ | NO T_P_ | / | N |
|  | North East | NO C_T_ | NO T_P_ | / | N |
|  | North East | NO C_T_ | NO T_P_ | / | N |
|  | North East | 37.09 | 27.32 | 1 to 50 | WP |
|  | North East | 36.94 | 22.39 | 1 to 50 | WP |
|  | North East | NO C_T_ | NO T_P_ | / | N |
|  | North East | 38.92 | 24.17 | 1 to 50 | WP |
|  | North East | NO C_T_ | NO T_P_ | / | N |
|  | North East | 36.92 | 23.36 | 1 to 50 | WP |
|  | North East | NO C_T_ | NO T_P_ | / | N |
|  | North East | 30.05 | 18.14 | 1 to 10^3^ | P |
|  | North East | 29.33 | 20.36 | >10^3^ | SP |
|  | North East | NO C_T_ | NO T_P_ | / | N |
|  | North East | 36.30 | 22.74 | 1 to 50 | WP |
|  | North East | NO C_T_ | NO T_P_ | / | N |
|  | North East | 35.67 | 22.73 | / | N |
|  | North East | NO C_T_ | NO T_P_ | / | N |
|  | North East | 27.94 | 17.20 | >10^3^ | SP |
|  | North East | NO C_T_ | NO T_P_ | / | N |
|  | North East | 28.51 | 16.97 | >10^3^ | SP |
|  | North East | 34.09 | 22.36 | 1 to 10^3^ | P |
|  | North East | 29.10 | 19.69 | >10^3^ | SP |
|  | North East | NO C_T_ | NO T_P_ | / | N |
|  | North East | 34.23 | 26.32 | 1 to 10^3^ | P |
|  | North East | 32.02 | 20.35 | 1 to 10^3^ | P |
|  | North East | 33.95 | 21.14 | 1 to 10^3^ | P |
|  | North East | 32.85 | 23.36 | 1 to 10^3^ | P |
|  | North East | NO C_T_ | NO T_P_ | / | N |
|  | North East | 33.68 | 22.17 | 1 to 10^3^ | P |
|  | North East | 32.87 | 23.21 | 1 to 10^3^ | P |
|  | North East | 35.69 | 20.36 | 1 to 50 | WP |
|  | North East | 31.88 | 18.94 | 1 to 10^3^ | P |
|  | North East | 35.07 | 24.36 | 1 to 50 | WP |
|  | North East | 32.89 | 21.01 | 1 to 10^3^ | P |
|  | North East | NO C_T_ | NO T_P_ | / | N |
|  | North East | NO C_T_ | NO T_P_ | / | N |
|  | North East | NO C_T_ | NO T_P_ | / | N |
|  | North East | 37.55 | 24.45 | 1 to 50 | WP |
|  | North East | NO C_T_ | NO T_P_ | / | N |
|  | North East | 32.88 | 19.69 | 1 to 10^3^ | P |
|  | North East | NO C_T_ | NO T_P_ | / | N |
|  | North East | 36.27 | 23.39 | 1 to 50 | WP |
|  | North East | 33.95 | 25.14 | 1 to 10^3^ | P |
|  | North East | 37.55 | 24.39 | 1 to 50 | WP |
|  | North East | NO C_T_ | NO T_P_ | / | N |
|  | North East | 37.29 | 28.41 | 1 to 50 | WP |
|  | North East | NO C_T_ | NO T_P_ | / | N |
|  | North East | NO C_T_ | NO T_P_ | / | N |
|  | North East | NO C_T_ | NO T_P_ | / | N |
|  | North East | 35.28 | 26.54 | 1 to 50 | WP |
|  | North East | 37.23 | 27.17 | 1 to 50 | WP |
|  | North East | NO C_T_ | NO T_P_ | / | N |
|  | North East | NO C_T_ | NO T_P_ | / | N |
|  | North East | NO C_T_ | NO T_P_ | / | N |
|  | North East | NO C_T_ | NO T_P_ | / | N |
|  | North East | NO C_T_ | NO T_P_ | / | N |
|  | North East | NO C_T_ | NO T_P_ | / | N |
|  | North East | NO C_T_ | NO T_P_ | / | N |
|  | North East | NO C_T_ | NO T_P_ | / | N |
|  | North East | 37.85 | NO T_P_ | 1 to 50 | WP |
|  | North East | NO C_T_ | NO T_P_ | / | N |
|  | North East | 36.61 | 22.68 | / | N |
|  | North East | NO C_T_ | NO T_P_ | 1 to 50 | WP |
|  | North East | NO C_T_ | NO T_P_ | / | N |
|  | North East | 34.29 | 21.14 | 1 to 50 | WP |
|  | South West | 37.34 | 23.14 | 1 to 50 | WP |
|  | South West | NO C_T_ | NO T_P_ | / | N |
|  | South West | 28.78 | 16.25 | >10^3^ | SP |
|  | South West | 37.32 | 20.36 | 1 to 50 | WP |
|  | South West | 29.97 | 18.36 | >10^3^ | SP |
|  | South West | 37.28 | 27.59 | 1 to 50 | WP |
|  | South West | NO C_T_ | NO T_P_ | / | N |
|  | South West | NO C_T_ | NO T_P_ | / | N |
|  | South West | 32.31 | 23.14 | 1 to 10^3^ | P |
|  | South West | 32.52 | 21.39 | 1 to 10^3^ | P |
|  | South West | NO C_T_ | NO T_P_ | / | N |
|  | South West | 30.87 | 18.62 | 1 to 10^3^ | P |
|  | South West | NO C_T_ | NO T_P_ | / | N |
|  | South West | 32.35 | 20.78 | 1 to 10^3^ | P |
|  | South West | NO C_T_ | NO T_P_ | / | N |
|  | South West | NO C_T_ | NO T_P_ | / | N |
|  | South West | NO C_T_ | NO T_P_ | / | N |
|  | South West | 34.30 | 20.67 | 1 to 10^3^ | P |
|  | South West | 31.44 | 19.39 | 1 to 10^3^ | P |
|  | South West | NO C_T_ | NO T_P_ | / | N |
|  | South West | NO C_T_ | NO T_P_ | / | N |
|  | South West | NO C_T_ | NO T_P_ | / | N |
|  | South West | NO C_T_ | NO T_P_ | / | N |
|  | South West | NO C_T_ | NO T_P_ | / | N |
|  | South West | NO C_T_ | NO T_P_ | / | N |
|  | South West | NO C_T_ | NO T_P_ | / | N |
|  | South West | NO C_T_ | NO T_P_ | / | N |
|  | South West | 37.27 | 26.35 | 1 to 50 | WP |
|  | South West | NO C_T_ | NO T_P_ | / | N |
|  | South West | NO C_T_ | NO T_P_ | / | N |
|  | South West | NO C_T_ | NO T_P_ | / | N |
|  | South West | NO C_T_ | NO T_P_ | / | N |
|  | South West | NO C_T_ | NO T_P_ | / | N |
|  | South West | NO C_T_ | NO T_P_ | / | N |
|  | South West | NO C_T_ | NO T_P_ | / | N |
|  | South West | NO C_T_ | NO T_P_ | / | N |
|  | South West | 37.76 | NO T_P_ | 1 to 50 | WP |
|  | South West | NO C_T_ | NO T_P_ | / | N |
|  | South West | NO C_T_ | NO T_P_ | / | N |
|  | South West | 37.79 | 26.04 | 1 to 50 | WP |
|  | South West | 37.52 | 24.93 | 1 to 50 | WP |
|  | South West | NO C_T_ | NO T_P_ | / | N |
|  | South West | 34.52 | 21.65 | 1 to 10^3^ | P |
|  | South West | NO C_T_ | NO T_P_ | / | N |
|  | South West | 36.11 | 22.87 | 1 to 50 | WP |
|  | South West | NO C_T_ | NO T_P_ | / | N |
|  | South West | NO C_T_ | NO T_P_ | / | N |
|  | South West | NO C_T_ | NO T_P_ | / | N |
|  | South West | 37.13 | 26.11 | 1 to 50 | WP |
|  | South West | NO C_T_ | NO T_P_ | / | N |
|  | South West | NO C_T_ | NO T_P_ | / | N |
|  | South China | NO C_T_ | NO T_P_ | / | N |
|  | South China | NO C_T_ | NO T_P_ | / | N |
|  | South China | NO C_T_ | NO T_P_ | / | N |
|  | South China | 37.57 | 22.17 | 1 to 50 | WP |
|  | South China | 37.27 | 26.44 | 1 to 50 | WP |
|  | South China | NO C_T_ | NO T_P_ | / | N |
|  | South China | NO C_T_ | NO T_P_ | / | N |
|  | South China | NO C_T_ | NO T_P_ | / | N |
|  | South China | NO C_T_ | NO T_P_ | / | N |
|  | South China | NO C_T_ | NO T_P_ | / | N |
|  | South China | NO C_T_ | NO T_P_ | / | N |
|  | South China | NO C_T_ | NO T_P_ | / | N |
|  | South China | 37.15 | 25.32 | 1 to 50 | WP |
|  | South China | NO C_T_ | NO T_P_ | / | N |
|  | South China | NO C_T_ | NO T_P_ | / | N |
|  | South China | NO C_T_ | NO T_P_ | / | N |
|  | South China | 35.94 | 25.13 | 1 to 50 | WP |
|  | South China | NO C_T_ | NO T_P_ | / | N |
|  | South China | 37.10 | 23.39 | 1 to 50 | WP |
|  | South China | NO C_T_ | NO T_P_ | / | N |
|  | South China | NO C_T_ | NO T_P_ | / | N |
|  | South China | NO C_T_ | NO T_P_ | / | N |
|  | South China | NO C_T_ | NO T_P_ | / | N |
|  | South China | NO C_T_ | NO T_P_ | / | N |
|  | South China | NO C_T_ | NO T_P_ | / | N |
|  | South China | NO C_T_ | NO T_P_ | / | N |
|  | South China | NO C_T_ | NO T_P_ | / | N |
|  | South China | NO C_T_ | NO T_P_ | / | N |
|  | South China | NO C_T_ | NO T_P_ | / | N |
|  | South China | NO C_T_ | NO T_P_ | / | N |
|  | South China | NO C_T_ | NO T_P_ | / | N |
|  | South China | 31.26 | 18.66 | 1 to 10^3^ | P |
|  | South China | NO C_T_ | NO T_P_ | / | N |
|  | South China | 36.69 | NO T_P_ | 1 to 50 | WP |
|  | South China | NO C_T_ | NO T_P_ | / | N |
|  | South China | NO C_T_ | NO T_P_ | / | N |
|  | South China | NO C_T_ | NO T_P_ | / | N |
|  | South China | NO C_T_ | NO T_P_ | / | N |
|  | South China | NO C_T_ | NO T_P_ | / | N |
|  | South China | NO C_T_ | NO T_P_ | / | N |
|  | South China | 37.17 | 23.47 | 1 to 50 | WP |
|  | South China | NO C_T_ | NO T_P_ | / | N |
|  | Central China | NO C_T_ | NO T_P_ | / | N |
|  | Central China | NO C_T_ | NO T_P_ | / | N |
|  | Central China | NO C_T_ | NO T_P_ | / | N |
|  | Central China | NO C_T_ | NO T_P_ | / | N |
|  | Central China | NO C_T_ | NO T_P_ | / | N |
|  | Central China | NO C_T_ | NO T_P_ | / | N |
|  | Central China | NO C_T_ | NO T_P_ | / | N |
|  | Central China | NO C_T_ | NO T_P_ | / | N |
|  | Central China | NO C_T_ | NO T_P_ | / | N |
|  | Central China | NO C_T_ | NO T_P_ | / | N |
|  | Central China | 36.75 | 24.93 | 1 to 50 | WP |
|  | Central China | NO C_T_ | NO T_P_ | / | N |
|  | Central China | 35.12 | 20.36 | 1 to 50 | WP |
|  | Central China | NO C_T_ | NO T_P_ | / | N |
|  | Central China | NO C_T_ | NO T_P_ | / | N |
|  | Central China | NO C_T_ | NO T_P_ | / | N |
|  | Central China | NO C_T_ | NO T_P_ | / | N |
|  | Central China | 34.75 | 26.48 | 1 to 10^3^ | P |
|  | Central China | NO C_T_ | NO T_P_ | / | N |
|  | Central China | NO C_T_ | NO T_P_ | / | N |
|  | Central China | 30.75 | 17.12 | 1 to 10^3^ | P |
|  | Central China | 34.61 | 25.96 | 1 to 10^3^ | P |
|  | Central China | NO C_T_ | NO T_P_ | / | N |
|  | Central China | 36.27 | 23.33 | 1 to 50 | WP |
|  | Central China | 37.92 | 29.31 | 1 to 50 | WP |
|  | Central China | NO C_T_ | NO T_P_ | / | N |
|  | Central China | NO C_T_ | NO T_P_ | / | N |
|  | Central China | 27.95 | 15.19 | >10^3^ | SP |
|  | Central China | 35.80 | 27.30 | 1 to 50 | WP |
|  | Central China | 30.73 | 19.91 | 1 to 10^3^ | P |
|  | Central China | 36.16 | 26.64 | 1 to 50 | WP |
|  | Central China | 33.36 | 20.03 | 1 to 10^3^ | P |
|  | Central China | NO C_T_ | NO T_P_ | / | N |
|  | Central China | 27.61 | 16.69 | >10^3^ | SP |
|  | Central China | NO C_T_ | NO T_P_ | / | N |
|  | Central China | NO C_T_ | NO T_P_ | / | N |
|  | Central China | 35.75 | 26.24 | 1 to 50 | WP |
|  | Central China | 37.71 | 23.30 | 1 to 50 | WP |
|  | Central China | 37.25 | 27.91 | 1 to 50 | WP |
|  | Central China | 35.07 | 20.09 | 1 to 50 | WP |
|  | Central China | 35.35 | 23.78 | 1 to 50 | WP |
|  | Central China | 36.54 | 26.84 | 1 to 50 | WP |
|  | Central China | 36.27 | 23.76 | 1 to 50 | WP |
|  | Central China | NO C_T_ | NO T_P_ | / | N |
|  | Central China | 36.12 | 20.93 | 1 to 50 | WP |
|  | Central China | 32.07 | 23.24 | 1 to 10^3^ | P |
|  | Central China | 35.32 | 22.90 | 1 to 50 | WP |
|  | Central China | 36.78 | 28.07 | 1 to 50 | WP |
|  | Central China | 32.96 | 25.51 | 1 to 10^3^ | P |
|  | Central China | NO C_T_ | NO T_P_ | / | N |
|  | Central China | 36.53 | 27.00 | 1 to 50 | WP |
|  | Central China | 37.33 | 28.64 | 1 to 50 | WP |
|  | Central China | 34.24 | 24.77 | 1 to 10^3^ | P |
|  | Central China | NO C_T_ | NO T_P_ | / | N |
|  | Central China | 35.03 | 20.94 | 1 to 50 | WP |
|  | Central China | 37.82 | 24.96 | 1 to 50 | WP |
|  | Central China | 34.34 | 21.12 | 1 to 10^3^ | P |
|  | Central China | NO C_T_ | NO T_P_ | / | N |
|  | Central China | 32.66 | 20.36 | 1 to 10^3^ | P |
|  | Central China | 35.36 | 23.01 | 1 to 50 | WP |
|  | Central China | 34.97 | 23.98 | 1 to 10^3^ | P |
|  | Central China | 36.80 | 23.14 | 1 to 50 | WP |
|  | Central China | 38.00 | 25.98 | 1 to 50 | WP |
|  | Central China | NO C_T_ | NO T_P_ | / | N |
|  | Central China | NO C_T_ | NO T_P_ | / | N |
|  | East China | 35.48 | 21.39 | 1 to 50 | WP |
|  | East China | NO C_T_ | NO T_P_ | / | N |
|  | East China | NO C_T_ | NO T_P_ | / | N |
|  | East China | NO C_T_ | NO T_P_ | / | N |
|  | East China | NO C_T_ | NO T_P_ | / | N |
|  | East China | NO C_T_ | NO T_P_ | / | N |
|  | East China | 38.16 | 25.07 | 1 to 50 | WP |
|  | East China | NO C_T_ | NO T_P_ | / | N |
|  | East China | 34.80 | 20.88 | 1 to 10^3^ | P |
|  | East China | NO C_T_ | NO T_P_ | / | N |
|  | East China | NO C_T_ | NO T_P_ | / | N |
|  | East China | 37.60 | NO T_P_ | 1 to 50 | WP |
|  | East China | 34.14 | 25.21 | 1 to 10^3^ | P |
|  | East China | 35.04 | 26.36 | 1 to 50 | WP |
|  | East China | 35.81 | 24.74 | 1 to 50 | WP |
|  | East China | NO C_T_ | NO T_P_ | / | N |
|  | East China | NO C_T_ | NO T_P_ | / | N |
|  | East China | 31.69 | 18.36 | 1 to 10^3^ | P |
|  | East China | 37.26 | 23.01 | 1 to 50 | WP |
|  | East China | 26.53 | 14.84 | >10^3^ | SP |
|  | East China | 37.43 | 23.00 | 1 to 50 | WP |
|  | East China | NO C_T_ | NO T_P_ | / | N |
|  | East China | 35.22 | 19.32 | 1 to 50 | WP |
|  | East China | NO C_T_ | NO T_P_ | / | N |
|  | East China | 37.49 | 22.99 | 1 to 50 | WP |
|  | East China | NO C_T_ | NO T_P_ | / | N |
|  | East China | NO C_T_ | NO T_P_ | / | N |
|  | East China | 35.50 | 26.30 | 1 to 50 | WP |
|  | East China | 36.58 | 21.73 | 1 to 50 | WP |
|  | East China | NO C_T_ | NO T_P_ | / | N |
|  | East China | NO C_T_ | NO T_P_ | / | N |
|  | East China | NO C_T_ | NO T_P_ | / | N |
|  | East China | NO C_T_ | NO T_P_ | / | N |
|  | East China | 37.61 | 23.60 | 1 to 50 | WP |
|  | East China | NO C_T_ | NO T_P_ | 1 to 10^3^ | N |
|  | East China | 37.79 | 27.29 | 1 to 50 | WP |
|  | East China | 34.95 | 21.32 | 1 to 10^3^ | P |
|  | East China | 37.83 | 29.31 | 1 to 50 | WP |
|  | East China | 32.88 | 22.17 | 1 to 10^3^ | P |
|  | East China | NO C_T_ | NO T_P_ | / | N |
|  | East China | 31.42 | 19.39 | 1 to 10^3^ | P |
|  | East China | 38.05 | NO T_P_ | 1 to 50 | WP |
|  | East China | NO C_T_ | NO T_P_ | / | N |
|  | East China | NO C_T_ | NO T_P_ | / | N |
|  | East China | NO C_T_ | NO T_P_ | / | N |
|  | East China | NO C_T_ | NO T_P_ | / | N |
|  | East China | 37.02 | 27.01 | 1 to 50 | WP |
|  | East China | NO C_T_ | NO T_P_ | / | N |
|  | East China | NO C_T_ | NO T_P_ | / | N |
|  | East China | 36.21 | 26.24 | 1 to 50 | WP |
|  | East China | 34.42 | 24.25 | 1 to 10^3^ | P |
|  | East China | 37.90 | NO T_P_ | 1 to 50 | WP |
|  | East China | NO C_T_ | NO T_P_ | / | N |
|  | East China | NO C_T_ | NO T_P_ | / | N |
|  | East China | NO C_T_ | NO T_P_ | / | N |
|  | East China | NO C_T_ | NO T_P_ | / | N |
|  | East China | NO C_T_ | NO T_P_ | / | N |
|  | East China | 35.19 | 22.36 | 1 to 50 | WP |
|  | East China | 36.90 | 26.32 | 1 to 50 | WP |
|  | East China | NO C_T_ | NO T_P_ | / | N |
|  | East China | 35.95 | 25.30 | 1 to 50 | WP |
|  | East China | 35.53 | 23.77 | 1 to 50 | WP |
|  | East China | 36.07 | 26.80 | 1 to 50 | WP |
|  | East China | NO C_T_ | NO T_P_ | / | N |
|  | East China | 34.67 | 21.09 | 1 to 10^3^ | P |
|  | East China | NO C_T_ | NO T_P_ | / | N |
|  | East China | 37.25 | 27.47 | 1 to 50 | WP |
|  | East China | NO C_T_ | NO T_P_ | / | N |
|  | East China | NO C_T_ | NO T_P_ | / | N |
|  | East China | NO C_T_ | NO T_P_ | / | N |
|  | East China | NO C_T_ | NO T_P_ | / | N |
|  | East China | NO C_T_ | NO T_P_ | / | N |
|  | East China | NO C_T_ | NO T_P_ | / | N |
|  | East China | NO C_T_ | NO T_P_ | / | N |
|  | East China | NO C_T_ | NO T_P_ | / | N |
|  | East China | 27.15 | 17.01 | >10^3^ | SP |
|  | East China | NO C_T_ | NO T_P_ | / | N |
|  | East China | NO C_T_ | NO T_P_ | / | N |
|  | East China | NO C_T_ | NO T_P_ | / | N |
|  | East China | NO C_T_ | NO T_P_ | / | N |
|  | East China | NO C_T_ | NO T_P_ | / | N |
|  | East China | NO C_T_ | NO T_P_ | / | N |
|  | East China | 32.29 | 19.96 | 1 to 10^3^ | P |
|  | East China | 28.34 | 18.36 | >10^3^ | SP |
|  | East China | NO C_T_ | NO T_P_ | / | N |
|  | East China | NO C_T_ | NO T_P_ | / | N |
|  | East China | 33.47 | 23.14 | 1 to 10^3^ | P |
|  | East China | NO C_T_ | NO T_P_ | / | N |
|  | East China | NO C_T_ | NO T_P_ | / | N |
|  | East China | NO C_T_ | NO T_P_ | / | N |

^a^ DNA estimated concentration (copies/µl) based on Taqman qPCR standard curve.

^b^ Clinical samples are classified into categories (cat.) based on Taqman qPCR C_T_ values; SP, strong positive; P, positive; WP, weak positive; N, negative.

C_T_：Cycle threshold.

T_P_：Time to positive.
