# Supplementary material for: Development and Clinical Validation of a Potential Penside Colorimetric Loop-Mediated Isothermal Amplification Assay of Porcine Circovirus Type 3
Source: Front Microbiol. 2022 Jan 12;12:758064. doi: 10.3389/fmicb.2021.758064 (PMC8790240; doi:10.3389/fmicb.2021.758064)
Supplement: Supplementary file 3 [file Table_3.DOCX]

**Table S3: Detection results of 120 pooled swine serum samples using both vLAMP and a direct detection method**

| **No.** | **Taqman qPCR C_T_** | **vLAMP T_P_**  **（with DNA isolation）** | **vLAMP T_p_**  **（without DNA isolation）** |
| --- | --- | --- | --- |
|  | 29.62 | 18.17 | 15.42 |
|  | 29.33 | 20.36 | 24.61 |
|  | 27.94 | 17.20 | 23.26 |
|  | 28.51 | 16.97 | 21.39 |
|  | 29.10 | 19.69 | 27.14 |
|  | 28.78 | 16.25 | 22.23 |
|  | 29.97 | 18.36 | 22.14 |
|  | 27.95 | 15.19 | 19.33 |
|  | 27.61 | 16.69 | 23.47 |
|  | 26.53 | 14.84 | 20.17 |
|  | 27.15 | 17.01 | 19.39 |
|  | 28.34 | 18.36 | 23.74 |
|  | 33.44 | 19.69 | 24.30 |
|  | 31.44 | 19.39 | 29.01 |
|  | 34.03 | 26.80 | 30.09 |
|  | 33.99 | 23.67 | 25.41 |
|  | 30.05 | 18.14 | 26.24 |
|  | 32.02 | 20.35 | 20.99 |
|  | 33.95 | 21.14 | No T_P_ |
|  | 32.85 | 23.36 | No T_P_ |
|  | 30.73 | 19.91 | 26.07 |
|  | 31.88 | 18.94 | 32.64 |
|  | 33.95 | 25.14 | No T_P_ |
|  | 34.30 | 20.67 | 27.79 |
|  | 35.07 | 20.09 | No T_P_ |
|  | 35.75 | 26.24 | 33.04 |
|  | 36.12 | 20.93 | 24.36 |
|  | 36.78 | 28.07 | No T_P_ |
|  | 37.33 | 28.64 | No T_P_ |
|  | 37.82 | 24.96 | 26.36 |
|  | 38.16 | 25.07 | 26.09 |
|  | 38.73 | 25.31 | No T_P_ |
|  | 35.88 | 25.14 | 32.55 |
|  | 36.50 | 23.17 | 28.46 |
|  | 36.07 | 23.63 | 26.37 |
|  | 35.07 | 20.09 | No T_P_ |
|  | No C_T_ | No T_P_ | No T_P_ |
|  | No C_T_ | No T_P_ | No T_P_ |
|  | No C_T_ | No T_P_ | No T_P_ |
|  | No C_T_ | No T_P_ | No T_P_ |
|  | No C_T_ | No T_P_ | No T_P_ |
|  | No C_T_ | No T_P_ | No T_P_ |
|  | No C_T_ | No T_P_ | No T_P_ |
|  | No C_T_ | No T_P_ | No T_P_ |
|  | No C_T_ | No T_P_ | No T_P_ |
|  | No C_T_ | No T_P_ | No T_P_ |
|  | No C_T_ | No T_P_ | No T_P_ |
|  | No C_T_ | No T_P_ | No T_P_ |

C_T_：Cycle threshold.

T_P_：Time to positive.
