# Supplementary figures and images for: Development and Clinical Validation of a Potential Penside Colorimetric Loop-Mediated Isothermal Amplification Assay of Porcine Circovirus Type 3
Source: Front Microbiol. 2022 Jan 12;12:758064. doi: 10.3389/fmicb.2021.758064 (PMC8790240; doi:10.3389/fmicb.2021.758064)

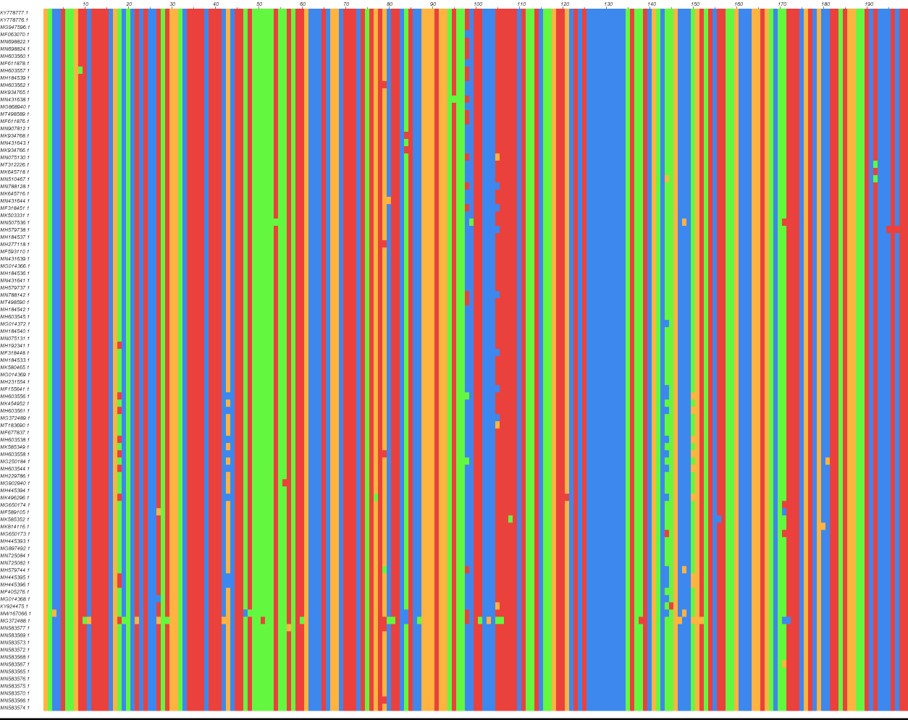

Supplement: Supplementary Figure 1 — Alignment of unique sequences retrieved from the blast query in NCBI (n = 97) with KY778777.1 as a reference for PCV3. The cap gene sequences covered by vLAMP primers was displayed. [file Image_1.JPEG]

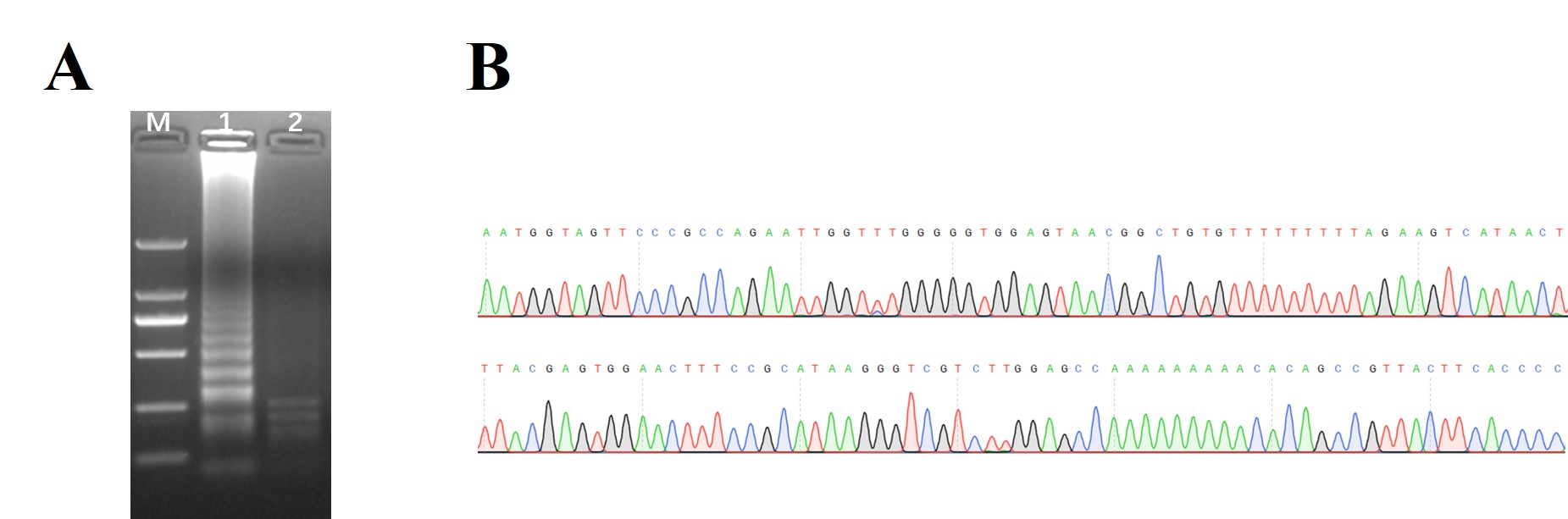

Supplement: Supplementary Figure 2 — Results of restriction endonuclease digestion and sequencing of vLAMP products. (A) Agarose gel electrophoresis shows vLAMP products after Eco47III digestion (lane 2), the expected band size after restriction endonuclease digestion is 140, 167, and 261 bp. Undigested vLAMP reaction product is also shown for comparison (lane 1). Lane M, DNA Marker DL-2000 (Takara). (B) Sequence Alignment shows the sequencing results of the 139 bp enzyme digestion products in A. The alignment indicated the identity was 97.12% (135/139), except for 4 nucleotides mismatching the reference sequences due to the sequencing error. [file Image_2.JPEG]

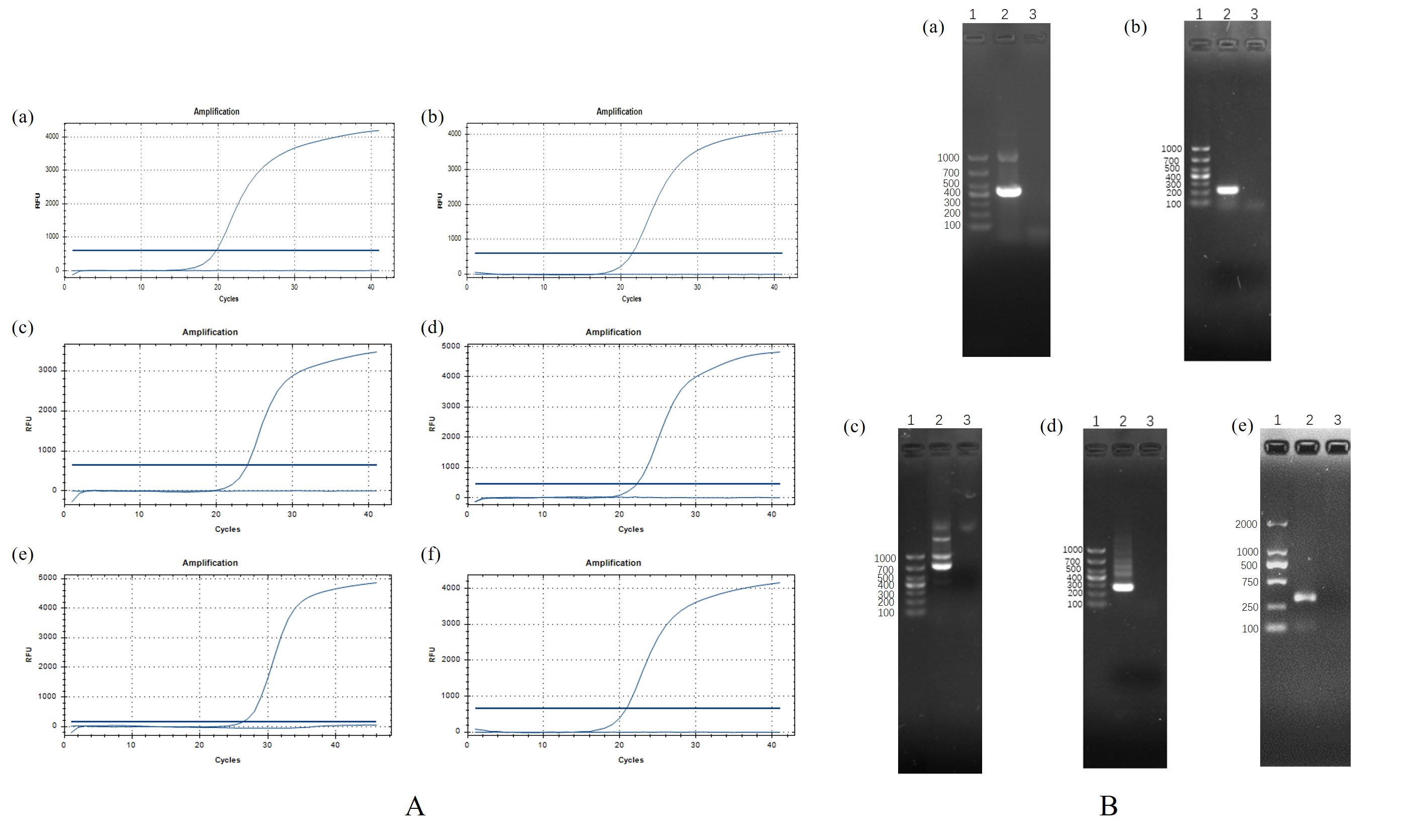

Supplement: Supplementary Figure 3 — Self-identification of CSFV, PRRSV, PRV, PEDV, JPEV, PCV1, PCV2, ASFV, PPV, and SIV. (A) Real-time qPCR experiments shows the self-identification results. The ddH2O was amplified as the NC in these detection. (a) JEPV, (b) PCV1, (c) PCV2, (d) ASFV, (e) PPV, (f) SIV. (B) Conventional PCR methods and agarose gel electrophoresis show the self-identification results. The expected band size of each virus are 400 bp (PRRSV), 217 bp (PRV), 671 bp (CSFV), 272 bp (CSFV) and 315 bp (PEDV), respectively. (a) PRRSV, (b) PRV, (c) and (d) CSFV, (e) PEDV. For each result, Lane 1, DNA marker; Lane 2, the PCR products; Lane 3, NC. [file Image_3.JPEG]

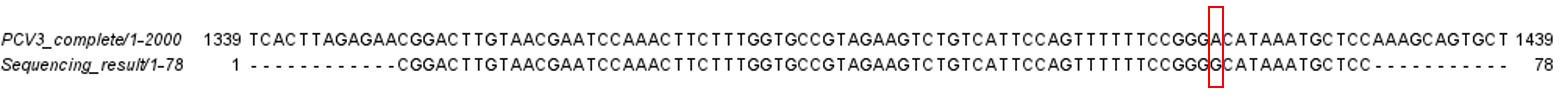

Supplement: Supplementary Figure 4 — Results of validation of the presence of PCV3 in samples (Ct > 32) by Taqman qPCR. The sequencing result of the 78 bp Taqman qPCR products using samples whose Ct > 32. The alignment indicated the identity was 98.7% (78/78), except for 1 nucleotides mismatching the reference sequences due to sequencing primer. [file Image_4.JPEG]
